# Risk Factors, Clinical Manifestations and Treatment Outcomes of Colon Cancer Patients in National Guard Hospital in Jeddah, Saudi Arabia

**DOI:** 10.7759/cureus.18150

**Published:** 2021-09-21

**Authors:** Sultan A Neazy, Zaher Mikwar, Aga S Sameer, Khalid Alghamdi, Hanin M Alowaydhi, Raghda T Hashim, Kamal H Salama

**Affiliations:** 1 Medicine, King Saud Bin Abdulaziz University for Health Sciences College of Medicine, Jeddah, SAU; 2 Surgical Oncology, King Abdulaziz Medical City, Ministry of National Guard Health Affairs, Jeddah, SAU; 3 Basic Medical Sciences, King Saud Bin Abdulaziz University for Health Sciences College of Medicine, Jeddah, SAU; 4 Quality Unit, King Saud Bin Abdulaziz University for Health Sciences College of Medicine, Jeddah, SAU; 5 Diagnostic Radiology, King Abdulaziz University Hospital, Jeddah, SAU

**Keywords:** colon cancer prevention, colon cancer and colon polyps, family history of colon cancer, chemotherapy, metastatic colo-rectal cancer

## Abstract

Introduction

Colon cancer is the third most common cancer worldwide and its incidence is increasing day by day. Provision of early management to cancer patients can lead to a good prognosis. Hence, we evaluated the risk factors, clinical manifestations and treatment outcomes for colon cancer patients in National Guard Health Affairs (NGHA), Jeddah, Saudi Arabia from January 2010 to December 2020 by comparing those results according to their age groups.

Methods

A retrospective cohort study was performed on 251 colon cancer patients who underwent a surgical procedure. The patients were divided into the following age groups: *≤* 50 (young), 51-60 and > 60 (old) years old. The demographic variables such as age and gender were collected. The results were classified into risk factors, clinical features and treatment outcomes. The comparison between different age groups was made using Chi-square or Fisher's exact test. The data was stored in Excel 2016 (Microsoft Corporation, Redmond, USA) and analyzed using SPSS (IBM Corp, Armonk, USA).

Results

The results revealed that most patients were males and the median age for diagnosis was 58 years old. There were 15.1% of patients with a positive family history. Moreover, the most common anatomical position was the left side of the colon in all age groups. Most patients had moderately differentiated colon cancer in the histopathological diagnosis. Laparotomy was the most common procedure done to patients in all age groups. There was no difference between all age groups and the aggressiveness of colon cancer. Young patients (*≤* 50 years) had a higher percentage to have 5-year recurrence rate (42 % vs 19% vs 25%, p-value < 0.05) in comparison to patients between 51-60 years and old patients (> 60 years) respectively. However, there was no association between all age groups and 5-year mortality rate (22% vs 9% vs 19%, p-value = 0.171).

Conclusion

In comparison to old patients (> 60 years), young patients (*≤* 50 years) have a more rate of recurrent colon cancer. In relation to all age groups, there were no differences in terms of the aggressive presentation or 5-year mortality rates. In addition, it appears that there were some differences between our study results and worldwide results. This may be because of occupational, cultural and/or genetic variations. Further studies with a higher number of patients and multicenter data collection are highly recommended.

## Introduction

Colon cancer is considered the third most common cancer around the world [1]. Recently, some studies showed that the incident rates are increasing in low-middle income countries with Asian countries being the highest in incidence rates [1]. In Saudi Arabia, colon cancer is the most common cancer in males and the second most common in females [2]. Moreover, the number of cases diagnosed with colorectal cancer in Saudi Arabia was 1347 cases in 2014 alone, which refers to the alarming increase in the incidence of colorectal cancer among Saudi nationals [3]. 

There are multiple risk factors reported for colon cancer which are: age, weight, body mass index (BMI) above 29.9, physical inactivity, chronic diseases (diabetes mellitus and hypertension), inflammatory diseases, low fiber diet, high fat diet, family history of colon cancer, inherited syndromes, alcohol intake, smoking, consuming red meat and processed product [4]. Moreover, another study revealed that chronic obstructive pulmonary disease (COPD) patients were at high risk for colon cancer [5]. Chromosomal instability, microsatellite instability and/or epigenetic changes in the DNA are pathways that can explain the pathophysiology of colon cancer [4]. Signs and symptoms of colon cancer include: change in bowel habits, blood in stool, abdominal pain or discomfort, anemia with unknown cause, weight loss and tiredness [4]. 

In Saudi Arabia, because of the early screening programs for adults older than 50 years old, the incidence and mortality rate has recently declined [6]. However, some studies found that colon cancer in patients younger than 40 years old has an increased incidence rate and aggression of the disease compared to patients older than 50 years [6,7]. In Saudi Arabia, there is an increase in young patients diagnosed with colon cancer in comparison to other populations in addition to low 5-year survival rates [8].

Moving to the clinical manifestations, a study revealed that younger patients were more likely to develop colon cancer on the left side in comparison to the elderly. In addition, most colon cancers were in stage 3 [6]. Focusing on the treatment outcomes, a study done in Saudi Arabia on colon cancer patients showed that the 3-year disease-free survival rate was 64.9% for laparoscopic colectomy and 55.3% following open colectomy [9]. Also, a study done in 2019 in the United States revealed that the combination of tumor resection and postoperative chemotherapy had a more positive effect on long-term survival rate compared with resection or chemotherapy alone [10].

To summarize, the risk factors, clinical manifestations and treatment outcomes play a major role in the different results of these studies. Although it is not recommended to screen young patients for colon cancer, there is a need to conduct such a uniform policy to improve the clinical practice in this age group [8]. This is because the age at diagnosis may play a role in the incidence and severity of the disease according to some articles [6,7]. Hence, a study in Saudi Arabia is recommended to determine the result of these measures. An early diagnosis and management do lead to a good prognosis, better quality of life and a possible cure [6,9]. Therefore, this study aims to identify and compare the risk factors, clinical manifestations and treatment outcomes between the young (≤ 50 years) and old (> 60 years) colon cancer patients in NGHA, Jeddah, Saudi Arabia.

## Materials and methods

A retrospective cohort study was conducted in the general surgery department in King Abdulaziz Medical City (KAMC), NGHA, Jeddah in the months of October to December 2020. The inclusion criteria involved all patients who were diagnosed with colon cancer between January 2010 to December 2020 by the histopathological report and underwent surgical intervention. In addition, it included all patients who were diagnosed with colon cancer by other departments such as gastroenterology and oncology. However, this study excluded the patients who had advanced (stage 4) colon cancer that can not be managed by surgical procedures. Moreover, it excluded the patients who were not fit for surgery due to general anesthesia issues.

At first, the total number of patients was 297. After applying the exclusion criteria, 46 patients were excluded and 251 patients remained. The data was collected with a 95% confidence interval and a 5% margin of error. The recommended sample size was 152 according to Raosoft software (Raosoft Inc, Seattle, USA) [11]. Because of the small recommended sample size, we conducted this study by doing a consecutive technique and all 251 patients were included. The data from January 2010 to November 2016 was gathered from the medical paper records. Moreover, the data from December 2016 to December 2020 was collected from the BESTCare system (internal automated medical records) (ezCaretech, Torrance, USA). We only were able to follow the patients which were diagnosed between January 2010 to December 2015 for 5 years (206 patients). On the other hand, 45 patients, who were diagnosed between January 2016 to December 2020, could not be evaluated for 5-years recurrence and mortality rates. 

The data collection sheet was composed of the following: family history, specific genes mutation, age, weight, inflammatory intestinal conditions, inherited syndromes, chronic disease, substance abuse, past history of cancers, histopathological grade, laterality, treatment outcome, stage, type of surgical intervention, pre/post-chemotherapy, pre/post-radiotherapy, lymph node yield, metastasis, recurrence rate and mortality. In order to compare these measures, patients were divided into different age groups as follows: ≤ 50, 51-60 and > 60 years old. All the patients' data obtained from either files or the BESTCare system was assessed by the data collection sheet variables mentioned above. The study was approved by King Abdullah International Medical Research Center (KAIMRC) with a study number of SP20/228/J. After the institutional review board (IRB) approval, the data was collected only by authorized members. However, some unavoidable factors did affect our studies like the small sample size and time frame of the study. 

The collected data was restored in the Excel program (Microsoft Corporation, Redmond, USA). After that, stored data were analyzed by the SPSS software (IBM Corp, Armonk, USA). The p-value of 0.05 was set as a threshold for statistical significance. Moreover, the data contained both qualitative, such as age and weight, and quantitative variables, such as gender and the histopathological subtypes. The frequency and percentage were used to analyze the qualitative data. Also, the qualitative data depended on the mean and standard deviation. The Chi-square test or Fisher's exact test were used to compare the risk factors, clinical features and treatment outcomes between the different age groups.

## Results

The results included 251 patients diagnosed with colon cancer and underwent surgical procedures between 2010 and 2020. Most patients were male (51.4%) and the median age of diagnosis was 58. The median (IQR) body mass index (BMI) was 26.7 (21.9-32.1). The chronic disease distribution among all patients was: 120 (47.4%) with diabetes, 112 (44.6%) with hypertension and 53 (21.1%) with dyslipidemia. In addition, only 42 (16.4%) of the patients were smokers and the mean packs per day in one year were two packs. There were 38 (15.1%) patients with a positive family history of colon cancer and only three (1.2%) with familial adenomatous polyposis (FAP). There was no relation between the mentioned risk factors and all age groups (p-value was insignificant).

Regarding the disease characteristics, most of patients had left-sided colon cancer in all age groups (64% of ≤ 50 years vs 56% of 51-60 years vs 51% of > 60 years, p-value = 0.293). Moreover, there were 51% (≤ 50 years) vs 55% (51-60 years) vs 50% (> 60 years) of patients who had a lympho-vascular invasion (p-value = 0.232). Colon cancer stages proportions were divided as follows: 3% (≤ 50 years) vs 9% (51-60 years) vs 16% (> 60 years) with stage 1, 41% vs 31% vs 29% with stage 2, 33% vs 36% vs 31% with stage 3 and 23% vs 23% vs 20% with stage 4 (p-value = 0.229). Among patients who were presented with metastatic tumors (M1), liver was the most common site of metastasis in all age groups (43% of ≤ 50 years vs 83% of 51-60 years vs 73% of > 60 years, p-value = 0.296), followed by lung (20% of ≤ 50 years vs 11% of 51-60 years of 14% of > 60 years, p-value = 0.651). The histopathological diagnosis revealed that most patients had moderate grade/differentiated colon cancer in all age groups (64% of ≤ 50 years vs 82% of 51-60 years vs 75% of > 60 years, p-value < 0.05). In addition, 34% of ≤ 50 years had a poorly differentiated tumor on the histopathological diagnosis in comparison to 18% of 51-60 years and 15% of > 60 years old patients (p-value < 0.05). Majority of patients underwent laparotomy (54% of ≤ 50 years vs 51% of 51-60 years vs 57% of > 60 years, p-value = 0.456). Other disease characteristics are demonstrated in Table 1.

**Table 1:** Comparing the disease characteristics and surgical procedure types between different age groups (n=251). Colon cancer T Stage, colon cancer N Stage, colon cancer Stage, disease laterality (anatomical position) and surgical procedure types.  *Fisher's exact test

Characteristics	Overall	Age group (years)			P-value
		≤ 50	51-60	> 60	
	N=251	N=61	N=78	N=112	
Patient-related n (%)					
Gender n (%)					
Female	122 (48.6)	18 (29.5)	39 (50)	65 (58)	< 0.05
Male	129 (51.4)	43 (70.5)	39 (50)	47 (42)	< 0.05
Disease-related n (%)					
Colon cancer T Stage n (%)					
Tis (carcinoma in situ)	2 (0.8)	0 (0)	0 (0)	2 (1.8)	<0.05*
T1 (grown into the Submucosa)	6 (2.4)	0 (0)	2 (2.6)	4 (3.6)	<0.05*
T2 (grown into the Muscularis Propria)	22 (8.8)	2 (3.3)	3 (3.8)	17 (15.2)	<0.05*
T3 (grown into the subserosa)	138 (55)	32 (52.5)	48 (61.5)	58 (51.8)	<0.05*
T4 (grown into the surface of the visceral peritoneum)	73 (29.1)	21 (34.4)	24 (30.8)	28 (25)	<0.05*
Unstated T stage (missing information)	10 (4)	6 (9.8)	1 (1.3)	3 (2.7)	<0.05*
Colon cancer N stage n (%)					
N0 (no lymph nodes involved)	112 (44.6)	25 (41)	33 (42.3)	54 (48.2)	0.232
N1 (1-3 lymph Nodes Involved)	73 (29.1)	15 (24.6)	28 (35.9)	30 (26.8)	0.232
N2 (More than 3 lymph Nodes Involved)	57 (22.7)	16 (26.2)	15 (19.2)	26 (23.2)	0.232
Unstated N stage (missing information)	9 (3.6)	5 (8.2)	2 (2.6)	2 (1.8)	0.232
Colon cancer stage n (%)					
Stage 0 (Tis, N0, M0)	4 (1.6)	0 (0)	1 (1.3)	3 (2.7)	0.229*
Stage 1 (T1-1, N0, M0)	27 (10.8)	2 (3.3)	7 (9)	18 (16.1)	0.229*
Stage 2 (T3-4, N0, M0)	81 (32.3)	25 (41)	24 (30.8)	32 (28.6)	0.229*
Stage 3 (T any N1, M0)	83 (33.1)	20 (32.8)	28 (35.9)	35 (31.3)	0.229*
Stage 4 (T any, N any, M1).	54 (21.5)	14 (23)	18 (23.1)	22 (19.6)	0.229*
Unstated (missing information)	2 (0.8)	0 (0)	0 (0)	2 (1.8)	0.229*
Disease laterality (anatomical Position) n (%)					
Right-sided of the colon	83 (33.1)	14 (23)	28 (35.9)	41 (36.6)	0.293
Left-sided of the colon	140 (55.8)	39 (63.9)	44 (56.4)	57 (50.9)	0.293
Both sides of the colon	28 (11.1)	8 (13.1)	6 (7.7)	14 (12.5)	0.293
Surgical procedure type					
Open surgery (laparotomy)	137 (54.6)	33 (54.1)	40 (51.3)	64 (57.1)	0.456*
Laparoscopic surgery	106 (42.2)	28 (45.9)	35 (44.9)	43 (38.4)	0.456*
Unstated (missing information)	8 (3.2)	0 (0)	3 (3.8)	5 (4.5)	0.456*

Focusing on the management and treatment outcomes, the median (IQR) pre-operative carcinoembryonic antigen (CEA) level was 4.1 (2-23) and the post-operative level was 2.5 (1.6-13.8). The chemotherapy distribution differed by age as follows: 18% (≤ 50 years) vs 14% (51-60 years) vs 12% (> 60 years) patients needed neo-adjuvant chemotherapy (p-value =0.805) and 64% (≤ 50 years) vs 57% (51-60 years) vs 42% (> 60 years) needed adjuvant chemotherapy (p-value < 0.05). Among the 251 colon cancer patients, 206 patients were followed for 5 years since they were diagnosed and managed in the period from January 2010 to December 2015. Among the following patients, there were 58% (≤ 50 years) vs 79% (51-60 years) vs 70% (> 60 years) of patients did not have recurrent cancer and 42% (≤ 50 years) vs 19% (51-60 years) vs 25% (> 60 years) had a recurrence rate within 5 years (p-value < 0.05). In addition, there were 22% (≤ 50 years) vs 9% (51-60 years) vs 19% (> 60 years) of patients died within 5 years due to cancer metastasis (p-value =0.171). Other treatment outcomes are shown in Table 2.

**Table 2:** Comparing the treatment outcomes between different age groups (n=206). Recurrence and mortality rates. *Fisher's exact test

Treatment outcome	Overall	Age group (years)			P-value
		≤ 50	51-60	> 60	
	N=206	N= 50	N=65	N=91	
Recurrence rate					
Yes (within 5 years)	56 (27.2)	21 (42)	12 (18.5)	23 (25.3)	< 0.05*
No	144 (70)	29 (58)	51 (78.5)	64 (70.3)	< 0.05*
Unstated (missing information)	6 (2.9)	0 (0)	2 (3.1)	4 (4.4)	< 0.05*
Mortality rate					
Yes (within 5 years)	34 (16.5)	11 (22)	6 (9.2)	17 (18.7)	0.171*
No	165 (80.1)	38 (76)	58 (89.2)	69 (75.8)	0.171*
Unstated (missing information)	7 (3.4)	1 (2)	1 (1.5)	5 (5.5)	0.171*

## Discussion

Talking about the risk factors, most patients in our study were male (51.4%) and the median age of diagnosis was 58 years. According to a study done in Germany, most patients were also males (53.9%). However, the median age of diagnosis was 71.7 years [12]. The number of patients with a positive family history of colon cancer was 38 (15.1%) patients. Likewise, a study done by Negri et al reported that 14.5% of patients have a positive family history of colon cancer [13]. There were no differences between the age groups in terms of all previously mentioned risk factors (p-value was insignificant). 

The incidence of left-sided colon cancer in both young (≤ 50 years) and old (> 60 years) was higher than right-sided colon cancer in our study. A Canadian study reported that young (≤ 40 years) were more likely to have colon cancer in the left side in comparison to old (> 60 years) patients [6]. On the other hand, an article published in Italy in 2021 showed variations based on age and gender where old female patients had a higher incidence of right-sided colon cancer [14]. Moreover, most of our young (≤ 50 years) and old (> 60 years) patients were diagnosed with stage 3 colon cancer with no association between them (34% vs 31%, p-value = 0.229). In contrast to our point, a study revealed that young ( ≤ 40 years) patients were more likely to have stage 3 colon cancer in comparison to old (> 60 years) patients [6]. Another literature review concluded that young (≤ 50 years) patients have a higher incidence to develop colon cancer at stage 3 than old (> 60 years) patients in multiple studies with the following percentages: 53% vs 41%, 62% vs 46% and 72% vs 63% [15]. This might indicate a lack of awareness of the importance of screening which was discussed in another article done in Saudi Arabia [2]. The majority of young and old patients underwent laparotomy in our study with no difference in the 5-year recurrence or mortality rates (p-value was insignificant). In contrast, a study done by Hakami et al revealed laparoscopic surgery was more than laparotomy with a higher percentage in the 3-year survival rate in patients with laparoscopic procedures (64.9% vs 55.3%) [9]. As mentioned in a systematic review done in Italy, laparoscopic procedures in colon cancer patients have a good prognosis and low rate of complications after the operation [16]. However, there is no difference in terms of 5-year survival rates in both types of surgeries [16].

Regarding management and treatment outcomes, there were 129 (51.4%) patients who needed adjuvant chemotherapy in our study. This result is like the Hakami et al study which reveals that 54.5% of the patients needed adjuvant chemotherapy [9]. Moreover, young patients were more likely to receive adjuvant chemotherapy in comparison to old patients in our study. This is similar to the study done in Canada revealing that chemotherapy was given more frequently to young patients (92% vs 57%, p-value < 0.05) [6]. As in our study, an article published in Sweden stated that the liver was the most common cause of metastasis in colon cancer followed by the lung [17]. Regarding liver metastasis, there were studies suggesting theories about how gastrointestinal tumors spread [18, 19]. Therefore, the findings in our study regarding metastasis go logically with the theories that suggest hematological metastasis happens through a portal system to the liver and then to the lung, which increases the risk when having lung metastasis to have concurrent liver metastasis. However, the metastasis can directly reach the lung through the lymphatic system [18]. The overall 5-year recurrence rate in our study was 27.2% and the 5-year mortality rate due to cancer metastasis was 16.5%. However, a study done in China revealed that the recurrence rate was 16.4% and the mortality rate was 12.9% [20]. This might be due to their early detection and regular follow-up of cancer in such patients. Moreover, the young patients were more likely to have a higher 5-years recurrence rate (42% vs 25%). This is opposite to a study done in Canada concluding that young patients had more chances of survival despite the aggressive presentation of the disease [6].

## Conclusions

To summarize, this study has identified and compared the possible risk factors, clinical features and management outcomes between the young (≤ 50) and old (> 60) colon cancer patients in NGHA, Jeddah, Saudi Arabia. This study concluded that young patients have a higher 5-years recurrence rate in comparison to old patients. In relation to all age groups, there were no differences in the aggressive presentation or 5-years mortality rates. Moreover, it appears that the results vary worldwide in terms of recurrence and mortality rates. This may be due to the difference in the levels of care available to the patients in different countries. However, this research has several limitations that affected the course of the study. One of these limitations is the small sample size. Another limitation is that there was some missing data about the patients' risk factors such as the type of diet (low fiber or high-fat diet). Additionally, 45 patients, who were diagnosed between January 2016 to December 2020, needed more time to be evaluated for 5-year recurrence and mortality rates, so they were not included in the follow-up. Further studies adding additional measurements with larger sample sizes and a multicentric approach are recommended.
